# A peptide-based anti-Adalimumab antibody assay to monitor immune response to biologics treatment in juvenile idiopathic arthritis and childhood chronic non-infectious uveitis

**DOI:** 10.1038/s41598-021-95920-9

**Published:** 2021-08-12

**Authors:** Hendrik Rusche, Edoardo Marrani, Feliciana Real-Fernandez, Roberta Ponti, Francesco Terzani, Ilaria Maccora, Olivier Monasson, Maria Vincenza Mastrolia, Elisa Peroni, Ilaria Pagnini, Rolando Cimaz, Anna Maria Papini, Gabriele Simonini, Paolo Rovero

**Affiliations:** 1 Peptlab@UCP Platform of Peptide and Protein Chemistry and Biology and UMR 8076 CNRS-BioCIS, CNRS, CY Cergy Paris Université, Neuville sur Oise, France; 2 Pediatric Rheumatology Unit, AOU Meyer, Florence, Italy; 3 Interdepartmental Laboratory of Peptide and Protein Chemistry and Biology, Department of NeuroFarBa, University of Florence, Sesto Fiorentino, Italy; 4 Department of Medicine, University Hospital of Udine, Udine, Italy; 5 Department of Clinical Sciences and Community Health, University of Milano, Milan, Italy; 6 Interdepartmental Laboratory of Peptide and Protein Chemistry and Biology, Department of Chemistry “Ugo Schiff”, University of Florence, Sesto Fiorentino, Italy; 7 Department of NeuroFarBa, University of Florence, Florence, Italy; 8 Present Address: Fischer analytics GmbH, Weiler, Germany

**Keywords:** Peptides, Biomarkers, Autoimmune diseases

## Abstract

Immune response to biologics treatment, while widely reported, yet fails to correlate with clinical outcomes and assay to assay comparison is often not possible. Hence, we developed a new peptide based-detection assay to stratify pediatric patients with juvenile idiopathic arthritis (JIA) or chronic non-infectious uveitis (CNU) and monitor anti-drug antibodies (ADAbs) formed as part of an immune response to treatment with the fully human monoclonal therapeutic antibody Adalimumab. Adalimumab derived synthetic peptides were optimized for maximum immunogenicity and were tested by SP-ELISA on a development cohort of 18 JIA and CNU treated patients. The two best performing peptides able to differentiate patient groups were selected for evaluation with a larger scale ELISA testing on a total of 29 sera from pediatric patients with JIA or CNU. The results of this peptide-based assay were compared to an in-house developed SPR biosensor ADAbs assay and a commercially available bridging ELISA. The first peptide, termed HC3, was able to positively detect ADAbs in 7 out of the 29 sera, while the second peptide, called LC3, was able to detect ADAbs in 11 out of 29 sera in the evaluation group. Following statistical data evaluation, it has been found that the detection of ADAbs using the peptide-based ELISA assay positively correlates with disease progression and remission. Two synthetic peptides derived from Adalimumab may provide a beneficial tool to clinicians for monitoring patient response to such treatment and taking informed decisions for treatment alternatives.

## Introduction

Juvenile idiopathic arthritis (JIA) is the most common rheumatic immune disease observed in children under the age of 16 years with an annual prevalence of 70.2/100,000^1^. Since TNF-α has been shown to play a key role in its pathogenesis^2^, treatment strategies to neutralize TNF-α have been developed, using monoclonal antibodies, chimeric fusion proteins or soluble proteins based on the TNF receptors. Adalimumab, a fully human TNF-α blocking monoclonal IgG developed and approved for several different autoimmune diseases, is one of the biologics most widely used in the treatment of JIA. Moreover, Adalimumab is currently also considered the most efficacious anti-TNF agent for childhood chronic non-infectious uveitis (CNU), isolated or associated with JIA^3^.

Although biologics-based therapeutic approaches show significantly improved outcomes^4^, up to 50% of patients treated with the TNF-α blocking chimeric monoclonal IgG Infliximab face primary failure, due to unresponsiveness of treatment or secondary failure as therapy efficacy is lost over time^1,5^. Indeed, Anti-Drug Antibodies (ADAbs) formation, as a consequence of biologics immunogenicity, has been associated with treatment failure and adverse effects, such as hypersensitivity reactions^6^. ADAbs are observed in patients treated with Adalimumab at a rate of up to 8% of patients after 8 weeks and up to 24% of patients after 60 weeks^7^ and it has been advocated that their presence may hamper treatment efficacy^8^.

Despite the similar structure and function of Adalimumab to human IgG1, anti-Adalimumab antibodies are reported in several inflammatory conditions^9–11^, including JIA^12^. Our group recently reported that the number of JIA activity relapses is associated with the presence of anti-Adalimumab antibodies^13^. The studies from Skrab-Baumgartner et al. and Brunelli et al. confirmed a correlation between detectable levels of ADAbs and higher disease activity^14,15^, an observation which has also been reported for patients with JIA associated uveitis^16^. However, two previously published studies failed to demonstrate such correlation in JIA^17,18^.

A direct comparison between these conflicting results is challenging due to different ADAbs detection assays, different observation times, and different types of study. Even though methods to detect ADAbs, such as Solid-Phase Enzyme-Linked-Immunosorbent-Assays (SP-ELISA), Radioimmunoassays (RIA), and biosensor-based methods, e.g. Surface Plasmon Resonance (SPR), have been developed^19,20^, each of the aforementioned methods has its own advantages and disadvantages. As such a standardized method to detect ADAbs in patients has not yet been identified, neither in adulthood nor in childhood auto-immune diseases. Furthermore, large biomolecules such as therapeutic proteins may present several epitope regions though not all of those epitopes potentially recognized by patients’ antibodies may relate to disease progression and therapy outcome. The use of selective peptides targeting specific disease or treatment related antibodies, and in particular ADAbs, has been reported as potentially more advantageous as monitoring tools^21,22^. Additionally, whilst ADAbs formation in treated patients is widely observed, their role is still debated among clinicians, since no clear conclusions regarding their impact in clinical care has been reached.

In this complex scenario, in order to develop an evidence-based treatment approach based on biologics therapy, it is of the utmost importance for clinicians to have reliable tools for the detection and characterization of ADAbs and to understand their exact role in clinical practice. Accordingly, in order to clarify the mode of action of ADAbs involved in JIA and to develop a faster and more reliable assay for ease of use by clinicians, we present a pilot study testing several selected Adalimumab derived peptides in order to identify the best performing candidate synthetic probes. To this purpose, we utilize epitope mapping data, peripheral blood mononuclear cell (PBMC) peptide recognition data, and available crystal structure data^23–25^, setting out to rationally design and synthesize a set of peptide probes for the development of a fast ELISA assay, which has been tested in a cohort of 18 JIA and CNU patients and 8 controls. Two candidate Adalimumab-derived peptide epitopes have been identified, which were further validated using a second cohort of 29 pediatric patients with JIA or CNU, to design an assay able to differentiate and stratify patients treated with Adalimumab in a medically relevant context.

## Results

### Rational peptide design

**Figure 1:**
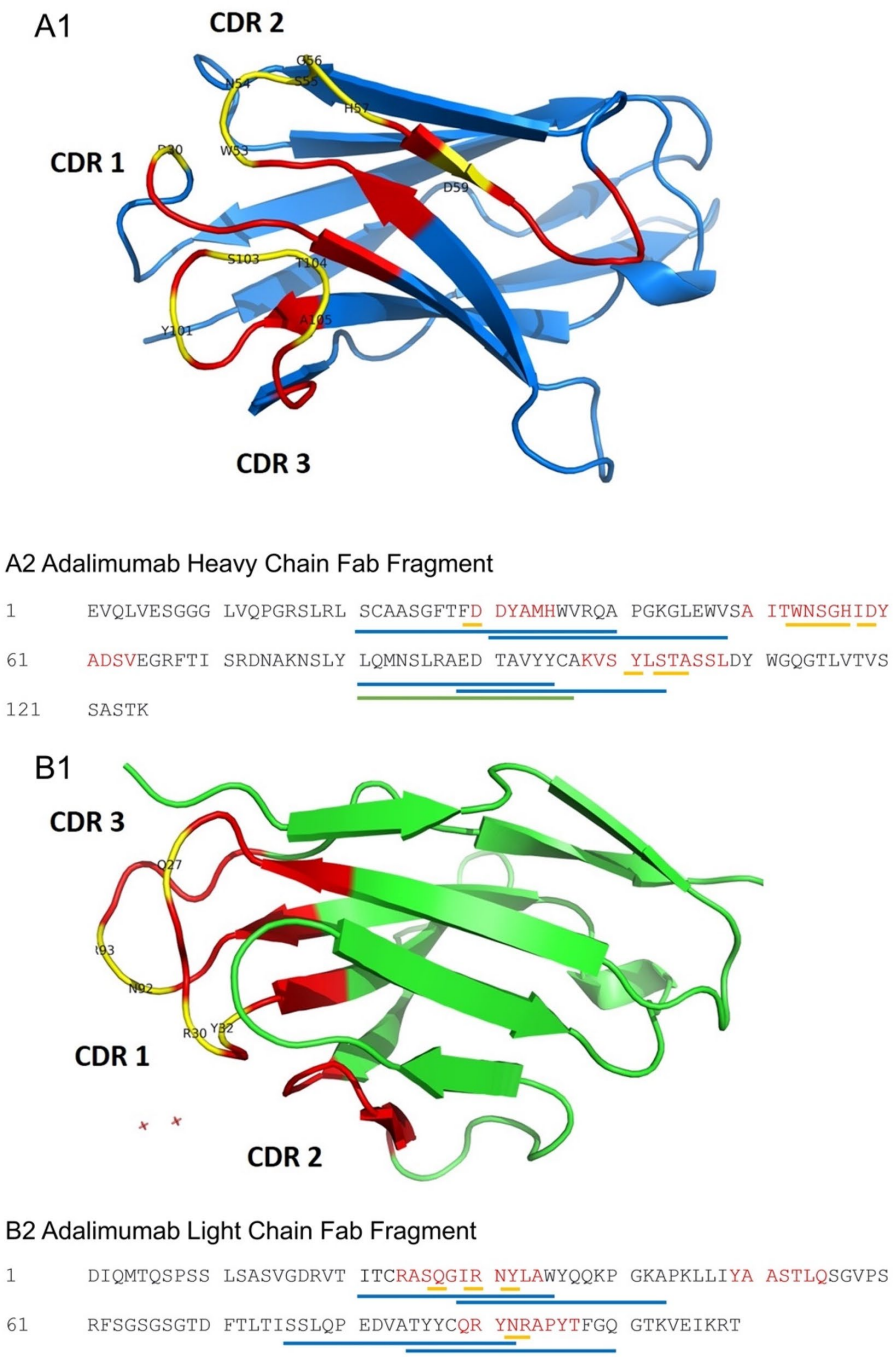
(**A1**) Crystal structure of Adalimumab heavy chain Fab fragment. (**A2**) Sequence map of Adalimumab heavy chain Fab fragment. Relevant candidate immunogenic peptide epitopes are reported. (**B1**) Adalimumab light chain Fab fragment. (**B2**) Sequence map. Underlined peptide fragments correspond to the same fragments reported in (A1). Different colors refer to the epitope identification methods used. Yellow: TNF-α contact points: TNF-α paratope was identified by crystal structure analysis of the antigen–antibody complex. Blue: epitope mapping by patient serum ELISA. Green: T-cell epitope mapping. Red: CDRs identified according to Kabat rules.

The primary amino acid sequence of Adalimumab was analyzed in order to assign the complementary determining regions (CDRs) following the Kabat rules^26^. This sequence-based analysis allows accurate assignment of the different CDRs on both heavy and light chains (Fig. 1, A2, B2). The paratope, made up of the CDRs, is comprised of a total of three CDRs per heavy and light chains, respectively. Peptides derived from these CDR regions should be good candidates as peptide probes. Van Schie et al. published in 2015 that antibody response against fully human and chimeric therapeutic antibodies, respectively Adalimumab and Infliximab, is primarily directed against the paratope, i.e. the antigen binding region of therapeutic antibodies^27^. In combination with the available crystal structure data (Fig. 1, A1, B1), which was published by Hu et al. in 2013 as a complex between the Adalimumab Fab fragment and TNF-α monomer, the contact points between target protein and antibody were identified (Fig. 1, A2, B2)^25^. Thus, inhibitory ADAbs would have to bind either directly or in close proximity to these contact points to sterically hinder complex formation and thus neutralizing the therapeutic effect of Adalimumab, causing the often observed loss of therapeutic response^25^. The epitope mapping data recently published by Homann et al., indicated a total of four epitope regions (Fig. 1, A2, B2) located on the Fv part of Adalimumab^23^. Epitope 1 on the heavy chain stretches from Ser-25 to Ile-58 and covers the CDR 1 as well as the TNF-α contact points as identified by Hu et al. The second heavy chain epitope is located close to CDR 3 and stretches from Leu-81 to Ser-103. The light chain epitopes are covering the CDRs 1 and 3, stretching from residues Ile-21 to Ala-43 and from Ser-77 to Gln-100, respectively. The epitope mapping data reported in the study of Homann et al. only found significant differences between patients and controls for the peptides covering the CDR-3 region on the heavy chain. The other epitopes were also recognized by control sera^23^. A PBMC epitope mapping was performed by Sekiguchi et al. in 2018 and the epitope region identified by overlapping Adalimumab peptides presented on MHC II clusters by PBMCs is indicated in green in Fig. 1, A2^24^. This kind of epitope mapping concerns the peptides presented to the immune system by PBMC cells and are thus considered antigenic. Clusters of peptides have been identified close to CDR 3 of the heavy chain. Patients and donors are not further disclosed in this work^24^. Considering all this available data, a total of nine peptide sequences were identified, synthesized, and tested by ELISA (Table 1).

**Table 1:** Adalimumab derived synthetic CDR region derived peptide sequences of the heavy chain (HC) and light chain (LC).

Peptide sequence overview	
Abbreviation	Sequence
HC1	^21^SCAASGFTFD**DYAMH**VVRQAA^40^
HC2	^50^**AITVNSGHIDYADSV**EGRFTI^70^
HC3	^95^YCAKVS**YLSTASSL**DYVGQG^114^
HC3.1	^81^LQMNSLRAEDTAVYYCAK**VSYLSTASSL**^108^
HC3.2	^85^SLRAEDTAVYYCAK**VS**^100^
HC3.3	^81^LQMNSLRAEDTAVYY^95^
LC1	^21^ITC**RASQGIRNYLA**VYQQKP^40^
LC2	^41^AlGKAPKLLIY**AASTLQS**GVPS^61^
LC3	^81^EDVATYYC**QRYNRAPYT**FGQ^100^

CDR regions assigned according to Kabat rules are highlighted in bold.

### Peptide synthesis

Peptides were synthesized by automated microwave assisted solid-phase peptide synthesis (SPPS) and purified by semi-preparative reverse phase HPLC (RP-HPLC) (for purification gradients see supplementary Table 1). Success of synthesis and purity were controlled by UPLC-MS. For immunological assays (ELISA), peptide purity of at least > 90% was used.

### Development of a peptide-based SP-ELISA assay for ADAbs detection

In order to identify the most suited Adalimumab derived synthetic peptide epitope, a first patients screening was performed by SP-ELISA, using as antigens the six HC peptides and the three LC peptides reported in Table 1. Sera from a selected group of 18 treated patients (sixteen affected by JIA and the remaining two affected by chronic idiopathic uveitis) and eight nontreated JIA patients, as control, were tested to identify the best performing peptides for further development. The main demographic and clinical characteristics of enrolled children at the time of sample collection are shown in Table 2. Disease duration ranged from 1 to 15 years among patients. Before starting Adalimumab administration, all patients were previously treated with methotrexate (MTX).

**Table 2:** Demographic and clinical data of JIA patient development and evaluation groups.

	Development group	Evaluation group
Number of patients (M/F)	18 (4/14)	29 (8/21)
Age (years) at inclusions (± SD)	10.9 (± 4.3)	10.1 (± 3.24)
Disease duration at study entry (years, ± SD)	4.6 (± 4.1)	4.7 (± 3.25)
Number of JIA patients (M/F)	16 (3/13)	25 (7/18)
**JIA category**		
Oligoarthritis	7	13
Polyarthritis	6	8
Psoriasis	1	2
Enthesitis-related arthritis	2	2
**Patients with uveitis: n (M/F)**	9 (1/8)	16 (3/13)
JIA associated Uveitis (M/F)	7 (0/7)	12 (2/10)
Chronic non-infectious uveitis (M/F)	2 (1/1)	4 (1/3)
ANA positivity (%)	11 (61%)	21 (72%)
Concomitant treatment with MTX (number)	86% (13)	58% (17)
Duration (months) of concomitant MTX (± SD)	32.2 (± 29.4)	40.6 (± 32.4)
Duration (months) of Adalimumab (± SD)	29.3 (± 32.7)	26.7 (± 28.2)
JIA inactive disease during drug administration (number/JIA patients)	69% (11/16)	64% (16/25)
Uveitis inactive disease during drug administration (number/uveitis patients)	78% (7/9)	87.5% (14/16^a^)
**For JIA patients**		
JADAS-10 (± SD) at Adalimumab introduction	13.7 (± 5.03)	14.8 (± 9.45)
JADAS-10 at *ADAbs* testing	2.7 (± 3.78)	2.8 (± 3.24)

^a^One patient with JIA was diagnosed with JIA-associated uveitis during treatment.

Among children with antinuclear antibodies (ANA) positivity, ten were JIA patients and one suffered from chronic idiopathic uveitis. Of note, two patients with JIA-associated uveitis had active uveitis, but no patients experienced a first onset of uveitis during the study period. Additionally, no patients experienced severe adverse events or injection related side effects. Samples from eight JIA patients, matched for age and gender, attending the Rheumatology Unit at Meyer Children Hospital and non-treated with Adalimumab were enrolled as controls.

All 18 patients treated with Adalimumab and 8 untreated JIA patients were tested for ADAbs detection using a homemade SP-ELISA based on the previously synthesized peptides as antigens, and the results were compared with those obtained using both a commercially available kit (LISA tracker, ref.: LTA 003-48, Theradiag, France) based on an bridging ELISA and an SPR-based method previously developed in our laboratory^19^.

**Figure 2:**
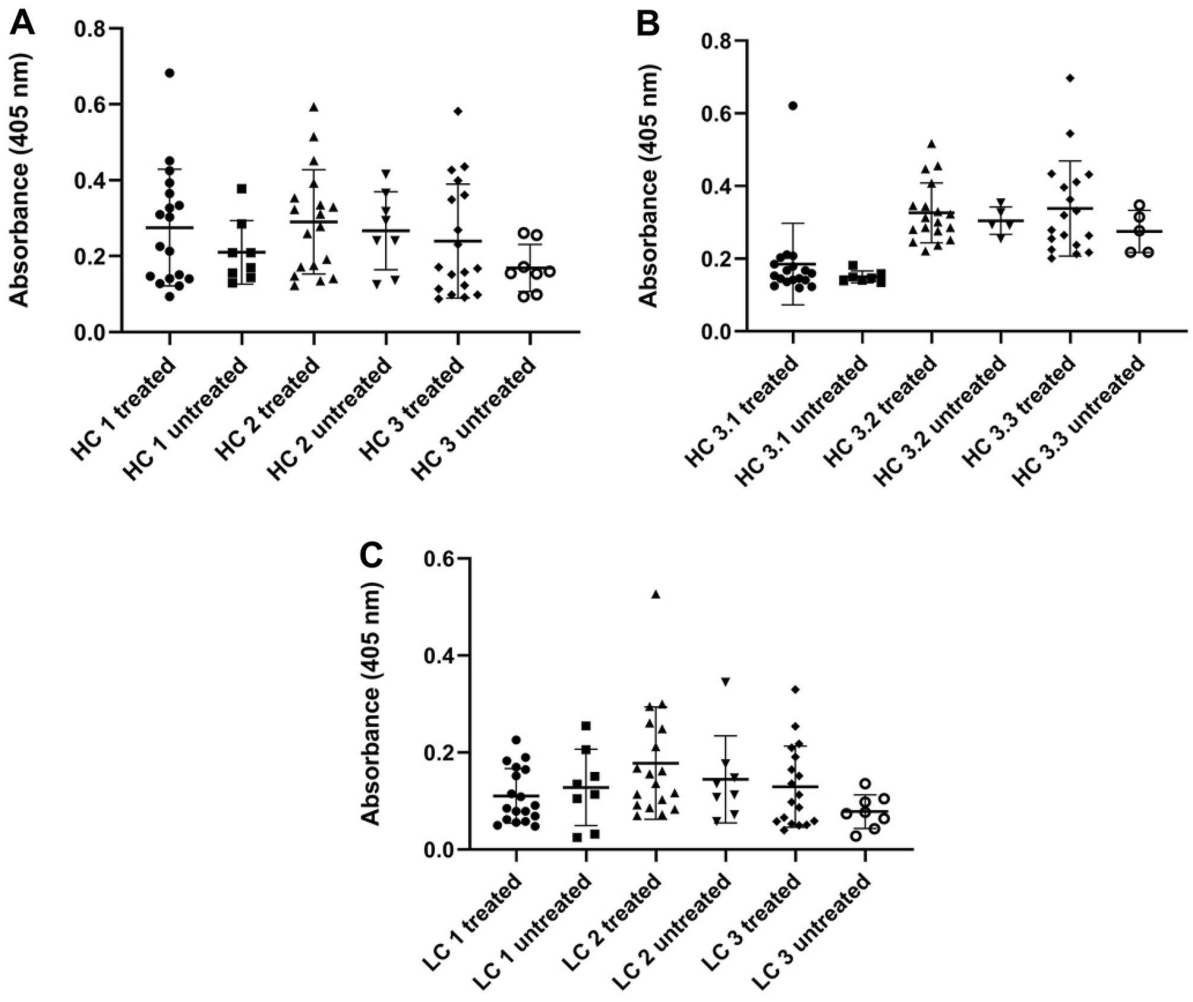
SP-ELISA of 18 sera of JIA patients treated with Adalimumab and 8 sera of untreated patients using CDR region Heavy Chain (HC) and Light Chain (LC) peptides as antigens (Table 1). Anti-Adalimumab antibodies were measured against: (**A**) HC 1–3 peptide antigens; (**B**) HC3.1–3.3 peptide antigens; (**C**) LC 1–3 peptide antigens. Significant differences in terms of absorbance between a group of treated patients and the negative controls is observed only for peptides HC3 and LC3 (*p* value HC3 = 0.0398, *p* value LC3 < 0.008).

The peptide-based SP-ELISAs classified patients as positive for anti-Adalimumab antibody formation if the absorbance value was higher than the mean value plus two times the standard deviation of the non-treated control sera. Results of the development group, summarized in Fig. 2, indicate that some treated JIA patients developed antibodies recognizing at least one of the peptides sequences derived from Adalimumab in ELISA, while patients of the control group did not have antibodies recognizing the peptides. As such, a low false positivity rate can be assumed for this assay.

In general, more patients showed Abs directed against peptides derived from the heavy chain: four patients positively recognized peptide HC1, three patients tested positive recognizing peptide HC2 and a total of seven out of 18 patients tested positive for peptide HC3 (Fig. 2A). Fewer patients tested positive for light chain derived peptides: no patients were recognizing LC1 while only one patient tested positive for Abs against LC2. Seven patients tested positive for Abs development against peptide LC3 (Fig. 2C). High agreement between Abs detection by HC3 and LC3 was observed: HC3 identified 71% of patients that were also identified by LC3. Lower agreement was observed between HC1 and HC3, as only three of the four positive HC1 patients were also positive as tested with HC3. Altogether eight patients resulted positive in at least one peptide-based SP-ELISA: five with JIA, two with JIA and chronic uveitis, and the remaining one with chronic idiopathic uveitis. The reference assays (LISA-tracker Theradiag, SPR) detected seven positive Adalimumab treated patients: five patients using the LISA tracker and six patients by SPR. Concomitant positivity for both tests was seen in four patients; one patient resulted positive only using the commercial kit, while two patients were positive only to SPR-based optical assay.

Comparing sensitivity among the three different performed assays in this patient cohort, only two out of seven patients (28%) who showed Abs detected using the reference assays were also positive with the peptide-based assay. Spearman correlation showed a low overall agreement between the peptide assay-based ELISA, LISA tracker kit, and SPR (*p* = ns). However, a statistically significant correlation has been found between ELISA and SPR-based assay (*r*_s_ = 0.62, *p* = 0.007).

Spearman correlation analysis showed that only Abs reacting to peptides HC3, HC2 and LC3 resulted in a statistically significant relation with the disease activity. In JIA-patients, presence of Abs to HC3 and LC3 peptides resulted in a strong correlation with the disease activity expressed as Juvenile Arthritis Disease Activity Score with 10 joint count (JADAS-10) score at the time of blood sample collection (*r*_s_ = 0.57, *p* = 0.02), as well as Abs reacting to HC2 (*r*_s_ = 0.63, *p* = 0.006), as reported in Table 3. A statistically significant inverse correlation has been observed between the presence of Abs recognizing HC3 and LC3 and inactive disease at the time of assay (*r*_s_ = 0.52, *p* = 0.004). That correlation has not been reproduced with HC2 (*r*_s_ =  − 0.31, *p* = 0.2). No significant correlation was observed between the presence of Abs and gender, JIA onset type, ANA positivity and disease duration. Of note, no significant correlation was found between presence of Abs and previous or concomitant treatment with MTX. Accordingly, peptides HC3 and LC3 were chosen for a further evaluation step.

**Table 3:** Pearson’s χ^2^ and Spearman’s correlation tests between disease activity (reported as JADAS-10 and JIA inactive disease) and anti-Adalimumab peptide antibodies with two-tailed *p*-values.

Pearson’s χ^2^ and Spearman’s correlation tests					
	Development group			Evaluation group	
	HC2	HC3	LC3	HC3	LC3
JADAS-10	0.630	0.570	0.570	0.420	0.460
Inactive, joint and eye disease, on drug	− 0.388	− 0.523	− 0.523	− 0.420	− 0.460
*p*-value	0.006	0.02	0.02	0.01	0.05
		0.004	0.004	0.01	0.005

A significant and positive correlation was observed with peptides HC2, HC3, and LC3 in the development group and peptides HC3 and LC3 in the evaluation group.

Peptides HC3.1, HC3.2 and HC3.3 detected no significant difference between treated patients and untreated controls (Fig. 2B) (t-test results for HC 3.1 *p* = 0.4552, no significant difference, HC3.2 *p* = 0.5779, no significant difference, HC3.3 *p* = 0.3139, no significant difference). These data indicate that these peptides are unable to differentiate between treated and untreated patients and did not detect ADAbs in the development group, unlike HC3. This may be attributed to the location of the CDR 3, which is located in the middle of HC3, while it is present in C-terminal position or not present at all in the peptides HC3.1–3.3, thus indicating the possible importance of the CDR 3 for antibody recognition.

### Further evaluation of selected peptides by SP-ELISA for ADAbs detection

As the initial screening proved to be promising for Adalimumab peptides HC3 and LC3, a larger scale testing was initiated using a total of 29 sera from Adalimumab treated JIA patients and eight non-treated JIA patients as controls. These patients are different from those of the development group: 25 affected by JIA, 12 of them with JIA associated uveitis, and 4 affected by CNU. The main demographic and clinical characteristics of enrolled pediatric patients at the time of sample collection are reported in Table 2. Mean disease duration was 4.7 years. All patients received MTX treatment before starting Adalimumab administration; 58% of patients were still receiving MTX at the time of the blood sample with wide variability in length of the exposure to drug. Among children with ANA positivity, 19 had JIA, and two CNU. One patient experienced a first onset of uveitis during Adalimumab administration: no patient experienced severe adverse events or injection related side effects.

**Figure 3:**
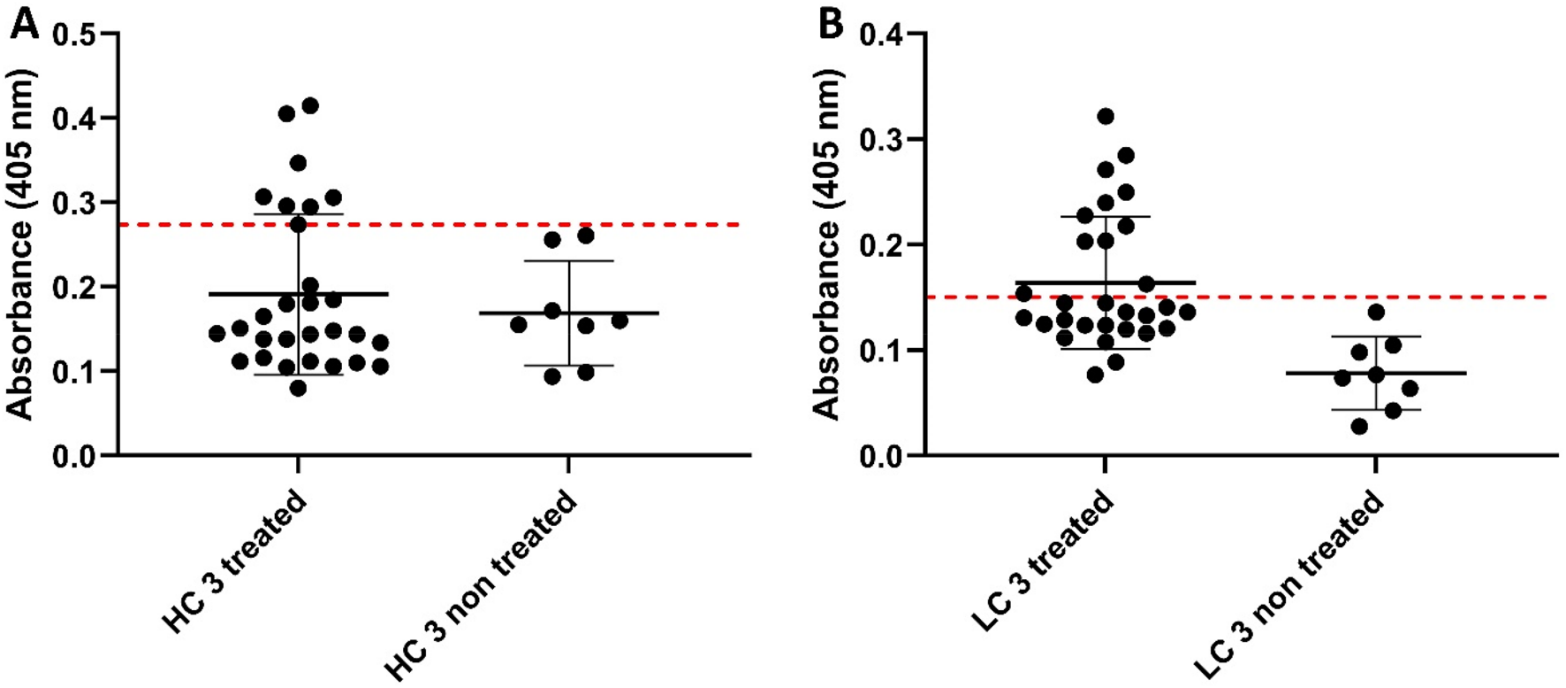
SP-ELISA to detect in patient sera anti-Adalimumab antibodies using: (**A**) peptide HC3 and (**B**) peptide LC3. Selected patients were Adalimumab treated JIA (n = 29) and non-treated JIA (n = 8). Conservative cut-off values were set by receiver-operator curve analysis (Fig. 1).

SP-ELISA scatter plot results are shown in Fig. 3, displaying treated vs non-treated patients. Cut-off values were calculated by ROC analysis for peptides HC3 and LC3 selecting 0.2845 [sensitivity 24% (95% CI interval 10.30% to 43.54%) and specificity 100% (95% CI interval 63.06% to 100.0%)] and 0.187 [sensitivity 31% (95% CI interval 15.28% to 50.83%) and specificity 100% (95% CI interval 63.06% to 100.0%)] respectively (see ROC curves in supplementary Fig. 1). Additionally, a titration experiment using HC3 and LC3 testing dilution of two treated JIA patients´ sera ranging from 1:50 to 1: 20,000 was performed (supplementary Fig. 3).

Both peptides found a statistically significant difference between treated and non-treated control patients (*p* value [HC3] = 0.0398, *p* value [LC3] < 0.0001) (Table 4). More importantly, analysis of the evaluation group revealed a significant correlation between HC3 and LC3 peptides and JADAS-10 score at the time of blood sample collection (*r*_s_ = 0.42, *p* = 0.01, and *r*_s_ = 0.46, *p* = 0.05, respectively), as reported in Table 3. We did not perform the analysis for uveitis activity since just 2 children at the time of the assay showed active uveitis (Table 2). Moreover, a statistically significant inverse correlation has been observed between the presence of Abs against HC3 and LC3 and, overall, inactive disease at the time of sample collection (*r*_s_ =  − 0.42, *p* = 0.01; and *r*_s_ =  − 0.46, *p* = 0.05, respectively). This statistical datum has been also kept when patients have been analyzed considering inactive disease for arthritis (HC3: *r*_s_ =  − 0.63, *p* = 0.001; LC3: *r*_s_ =  − 0.53, *p* = 0.01) and uveitis (HC3: *r*_*s*_ =  − 0.47, *p* = 0.01; LC3: *r*_s_ =  − 0.39, *p* = 0.05) independently.

**Table 4:** Spearman correlation (*r*_s_) between different assays and t-test to evaluate significant differences between the evaluation group of Adalimumab treated JIA patient sera (n = 29) and non-treated JIA patient sera (n = 8) (*p* value) including number of positively identified patients (+).

Spearman correlation									
Method		SP-ELISA				Bridging ELISA		Biosensor (SPR)	
Antigen		Adalimumab HC (95–114)		Adalimumab LC (81–100)		Modified Adalimumab (commercial)		Adalimumab	
Name		HC3		LC3		LISA tracker		SPR	
HC3		–		–		–		–	
LC3		0.8353		–		–		–	
LISA tracker		− 0.08068		− 0.07028		–		–	
SPR		0.2615		0.2114		0.07050		–	
**t-Test between treated and non-treated patients**									
*p* value		0.0398		< 0.0001		0.3267		< 0.0001	
(+) Patients		7		11		3		9	
**Agreement between assays**									
HC3 vs LC3	LC3 vs HC3		HC3 vs LISA tracker		LC3 vs LISA tracker		LISA tracker vs SPR		HC3/LC3 vs SPR
63.6% (7 of 11)	100% (11 of 7)		0% (0 of 7)		9.1% (1 of 11)		11.1% (1 of 9)		55.5% (5 of 9)

Overall agreement between the different assays: number of overlapping positively identified patients is reported.

Again, no statistically significant relation was observed for the other indicated clinical variables. Of note, the cumulative months of MTX administration, as mean ± SD, between children showing HC3 and LC3 peptides and children who did not show, were not different (HC3: 32.1 ± 31.5 vs 39.3 ± 27,8, *p* = 0.49 LC3:33.6 ± 31.9 vs 35.2 ± 28.1, *p* = 0.88). The presence of HC3 and LC3 was not associated with the MTX administration (χ^2^ = 0.63 and 0.29, respectively).

**Figure 4:**
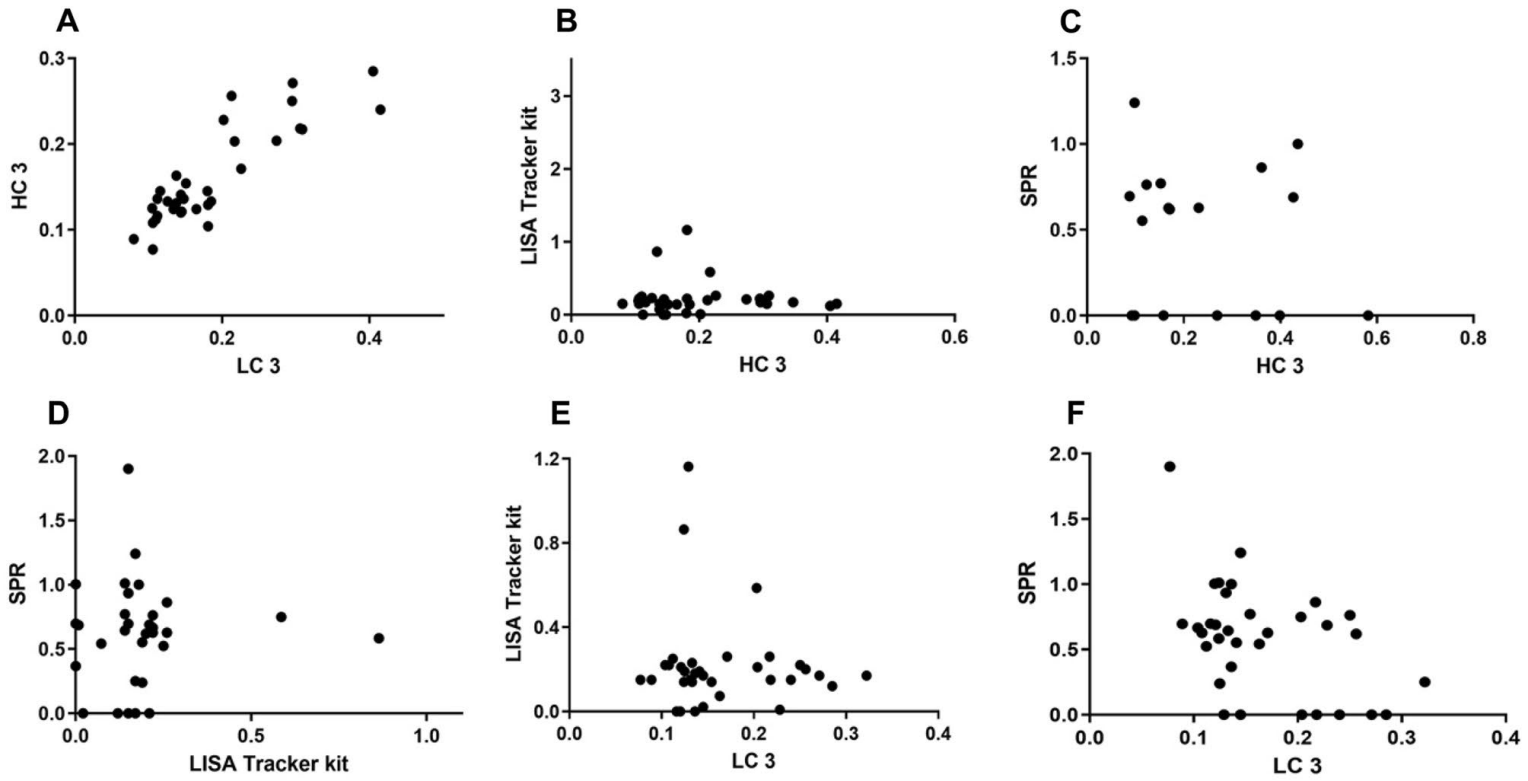
Scatter plot of HC3 and LC3 vs LC3/HC3, LISA Tracker and SPR results, respectively. Plot (**A**) shows the scatter plot of HC3 plotted against LC3. The diagonal distribution of plotted points show that the SP-ELISA results are in good agreement with both tested peptides. (**B**) and (**E**) Show the results of HC3 and LC3 plotted vs the LISA tracker kit, respectively. No clear correlation can be drawn from the diagram, indicating no correlation in detection of ADAbs. (**C**) and (**F**) Display the plot of HC3 and LC3 vs the SPR results. No clear correlation can be observed. (**D**) Displays the correlation between SPR and LISA tracker kit.

The same group of patients was also tested for Abs detection using the LISA-tracker kit from Theradiag (France). This commercially available kit recognizes patient Abs through immobilized Adalimumab. Presence of Abs is detected using enzyme-conjugated Adalimumab, which binds to Abs captured by the immobilized Adalimumab, enabling their detection. In the cohort of 29 sera tested, the LISA tracker found three patients positive compared to the seven and eleven patients found positive using the peptides HC3 and LC3, respectively. The statistical evaluation is summarized in Table 4: T-Test evaluation showed that the LISA tracker did not detect a significant difference between treated and non-treated patients (*p* = 0.3267). While both HC3 and LC3 peptides are in good agreement and identify the same sub-groups of patients as positive (Fig. 4A), there is little to no overlap as to which patients are considered positive by the LISA tracker kit and the synthetic peptides (Fig. 4B–E). In fact, from the scatter diagram of plotting HC3 vs LC3, a clear positive correlation between the two data sets is visible, while no positive correlation can be observed between any other two methods. Spearman correlation analysis found a high correlation (*r*_s_ = 0.8353) between HC3 and LC3, while only a negative agreement between the LISA tracker and HC3 (*r*_s_ =  − 0.08068) and LC3 (*r*_s_ =  − 0.07028) was observed. Thus, based on the correlation analysis it can be reasoned that the kit and the peptides recognize different groups of Abs found in patients treated with Adalimumab, i.e., Abs directed against different epitopes on the Adalimumab molecule. Most importantly, these different Ab families are endowed with diverse Adalimumab neutralization properties and therefore affect differently the clinical outcome of the treatment. However, as mentioned in the introduction, it should be considered that the comparison among different ADAbs detecting assays is hampered by several factors, including the lack of a standardized method and the inherently different architecture of each assay. Abs were also measured by SPR, as previously described by our group^19,20^, showing that five sera were detected positive for anti-Adalimumab Abs and four were detected borderline positive using this method. Statistical evaluation indicates that there is a significant difference between treated patients and non-treated patients (*p* < 0.0001). Spearman correlation showed a low agreement between the detection of Abs by SPR and by peptide-based ELISA; HC3 showed a correlation of *r*_s_ = 0.2615 and LC3 *r*_s_ = 0.2114 (Fig. 4C, F). Comparison between the LISA tracker kit and SPR also showed a low correlation *r*_s_ = 0.07050.

Finally, to demonstrate that the epitope represented by the peptides is indeed shared by the parent protein, i.e., Adalimumab, we performed an inhibition ELISA experiment with a representative patient serum, positive in both the peptide-based ELISA and the two reference assays, showing that ADAbs binding to peptide HC3 on the ELISA plate is 64% inhibited by Adalimumab in solution (supplementary Fig. 2). Interestingly, peptide LC3 in solution also inhibits the ADAbs binding to HC3 on the plate, suggesting that the two peptides may be part of the same discontinuous epitope.

## Discussion

The introduction of therapeutic monoclonal antibodies has been considered a turning point in the care of patients in the field of pediatric rheumatology. However, the repeated administration of monoclonal antibodies, with few exceptions, should be considered as an immunizing event. In subjects receiving anti-TNF-α agents, a vaccine-like response has been documented with strong humoral response and production of antibodies targeting the drug^28^. Thus, immunogenicity represents a negative effect of long-term treatment with anti-TNF-α modulators or blockers^29^. Despite the high percentage of patients reaching a controlled disease stage, discontinuation of anti-TNF-α agents due to ineffectiveness or adverse reactions is still a frequent event. Two mechanisms have been suggested for how ADAbs are able to reduce treatment efficacy. First, neutralizing ADAbs (ntADAbs) might be developed; these antibodies bind to pharmacologically active site of the biologic agent and might directly prevent its binding to the TNF-α. ADAbs that recognize other epitopes on the drug that do not affect the binding activity of the drug towards the therapeutic target, are called non-neutralizing ADAbs or binding ADAbs. Despite that ntADAbs are generally considered to be more relevant in the clinical setting, both ntADAbs and non ntADAbs can result in the formation of immune complexes by binding to the drug, thus increasing drug clearance and lowering the effective drug concentration. Till now, studies conducted on ADAbs in JIA patients produced contradictory results, not supporting routine clinical use of therapeutic drug monitoring and ADAbs measurement^10^; however, the different assays used hampered a meaningful comparison of the results and previous studies examined only the prevalence of ADAbs and no one until now evaluated the neutralizing activities of such antibodies^9^.

As stated above, different methods to detect ADAbs have been developed (i.e. ELISA, RIA, SPR, and others)^19,20^ and each method has its own advantages and disadvantages. ELISA are robust and fast assays, able to screen large number of patients at once with good reproducibility and high sensitivity at low cost. However, as we showed in a recent comparative study, only high affinity antibodies are detected by SP-ELISA in Adalimumab treated rheumatoid arthritis patients^20^. Improved “bridging” ELISAs for detection of ADAbs are commercially available, however they are unable to differentiate between neutralizing Fv directed ADAbs and non-neutralizing ones^30^. As such, these assays are unable to detect clinically relevant ADAbs. This problem is mainly due to the antigen selection for ADAbs identification, as also observed and frequently reported for other diagnostic technologies.

Biosensor based detection assays have the draw-back of requiring expensive equipment and offer lower throughput, as compared to ELISA assays, while at the same time they are more sensitive, when it comes to detection of different antibodies such as high and low affinity antibodies. RIA, whilst being less prone to Adalimumab level underestimation through inhibition of free Adalimumab or TNF-α present in the patient sample, are limited due to the special sample handling and equipment required for managing radioactivity-based detection assays. In general, there are differences in detection of neutralizing ADAbs, which appear to be more relevant in the clinical setting.

Thus, we set out to design an Adalimumab derived peptide as antigenic probe, to be used in ELISA allowing improved ADAbs detection. Utilizing bioinformatic tools and approaches, a total of six new peptide sequences were derived from Adalimumab and three peptides were chosen from literature and used in an initial development cohort of patients for screening. Sequences were selected to exhibit maximum immunogenicity: peptides were chosen from CDR regions of Adalimumab combining different pieces of information, i.e., sequence analysis using Kabat rules, crystal structure analysis, T-cell epitope mapping, and classical epitope mapping data. As such, a simplified epitope mapping could be performed allowing for a faster and more economical development of peptides in comparison to large proteins and glycoproteins, while at the same time displaying lower heterogeneity of the final product.

Although the different bioinformatic and experimental approaches used to identify epitopes produced different results, we observed a clear consensus, indicating the presence of relevant epitopes in overlapping portions of the CDR regions. These differences in epitope recognition may be explained by epitope spreading and migration: during somatic mutations, the epitope specificity of antibodies might change over time as part of the human immune response leading to clinically relevant epitopes mimicking close related epitopes^31^. Thus, mutations in the antibody variable regions can lead to changes in the epitope recognition of the antibodies, hence recognizing slightly different epitopes close in terms of topology to the immuno-dominant epitope. This suggests a possible immunodominant epitope located in close proximity to CDR 3 in both heavy and light chains, as indicated by the results of previous studies, showed in Fig. 1. This increased immunogenicity may be explained by higher proteolytic susceptibility, in turn leading to an increase in PBMC epitopes and consequently ADAbs formation directed against this part of Adalimumab. These peptides, derived from the variable Fv region, lack the sometimes-immunogenic glycosylated Fc part of full-size Adalimumab, thus allowing for the detection of only neutralizing antibodies, which recognize the paratope to TNF. Furthermore, the peptides represent a much smaller antigen compared to full length Adalimumab. As such, patient ADAbs recognizing discontinuous epitopes will bind either with reduced or too low affinity to the peptides, thus making observation of these ADAbs difficult. In fact, the positive correlation between HC3 and LC3 indicates a strong likelihood of a subset of ADAbs recognizing both peptides and thus may share the same discontinuous epitope, as suggested also by our inhibition ELISA experiment, showing cross-reactivity between the two peptides (supplementary Fig. 2).

When correlating the results of the peptide-based test with clinical outcome in our cohort, ADAbs directed against HC3 and LC3 peptides appeared a promising tool to monitor treatment in such patients. In our cohort Abs directed towards at least one of the HC3 and LC3 were found in 21/47 patients with a prevalence of 44%. The prevalence in our cohort is higher than the pooled prevalence of 21.5% from the meta-analysis by Doeleman et al.^12^ but close to the prevalence reported by our group in a previous report^14,16^ and by other authors, as well^12,15^. This higher prevalence might be explained by a higher sensitivity of our assay in comparison to the methods previously used. Moreover, the different observation times and different types of study do not allow a direct comparison between these studies. When analyzing data on length of exposure to Adalimumab in our cohort, we could not find any statistically significant correlation between length of treatment with Adalimumab and prevalence of ADAbs. However, in the longitudinal study by Brunelli et al. a significant increase in the prevalence of ADAbs was observed after 3 months of treatment with Adalimumab and was persistently stable till the end of the follow-up (24 months). Thus, ADAbs production seems to be an early event after starting of Adalimumab treatment and this observation could explain the absence of correlation in our cohort despite the wide range of exposure to Adalimumab, varying between 2 and 116 months (data not shown).

Of utmost importance, we found a statistically significant association between ADAbs directed toward at least one of the HC3 and LC3 probes and clinical response to treatment. We found that in both the development and evaluation cohorts the ADAbs positive patients had a statistically significant lower chance to present an inactive disease at the time of ADAbs determination. We also found a higher disease activity (defined as a higher JADAS10 score at the sample collection) in the ADAbs positive patients. This is in line with the results already published by our group^27^ and the data from the prospective study from Brunelli et al.^15^ and Skrabl-Baumgartner et al.^14^. We were not able to find an association with patients with active uveitis, since just two children had active uveitis. However, performing a sub-analysis related to number of patients exhibiting inactive uveitis, we identified a strong inverse correlation with the presence of antibodies. This is in accordance with the study conducted by Skrabl-Baumgartner et al. In their longitudinal study, 8/20 patients showed a loss of response to Adalimumab and active uveitis was associated with a persistent high level of ADAbs during the follow-up^16^. According to this study design, regular assessment of Adalimumab levels and ADAbs was performed after 1 and 3 months of treatment and every 3 months there-after. Alternatively, our study is an observational time-point study and thus our peptide-based assay should be validated in a prospective cohort of patients before reaching a definitive conclusion.

Interestingly, ADAbs detected by the other peptide-based assays and commercial bridging ELISA or the SPR-based optical assay did not show any clinical correlation in our present cohort, suggesting also from a clinical point of view that the HC3 and LC3 probes might selectively identify the ADAbs with a neutralizing action. Thus, according to the results of our test, we hypothesize that ADAbs directed against Adalimumab are not all equal in terms of biological significance and thus determination of ntADAbs might be of utmost importance in driving therapeutical decision. However, several limitations of our study need to be highlighted. First, this is only a cross-sectional pilot study conducted on a small population to identify the most promising peptide-based assays for a further evaluation in larger cohort of patients. As a second point, we did not perform monitoring of anti-TNF drug level in our patients; thus, we are not able to correlate ADAbs found by using peptide-based assays to serum Adalimumab trough level. However, it has been documented elsewhere that some patients with ADAbs in response to Infliximab still maintain prolonged remission despite serial measurements of high ADAbs and low Infliximab trough levels^32^. This in-vivo finding might suggest that ntADAbs acting through a competitive binding to the active sites of the anti-TNF agents may exert most of the inactivating action while the increased clearance of the drugs might play only a subsidiary role. Third, as a limitation of a time-point observational study, we could not evaluate the rate of loss of response to treatment or the need of switching biologic agents on a longer follow-up time. Moreover, we failed to show any association between ADAbs and exposure to MTX. This is in contrast with previous reports from literature, based on adults and children affected by chronic rheumatic disorders^10,24^. In these studies, MTX was found to correlate indirectly with ADAbs and concomitant treatment with such agent seems able to significantly reduce the immunogenicity of anti-TNF agents and so the loss of response^12^. It is possible that our study is underpowered to test this potential difference. Additionally, being a cross-sectional retrospective study, our analysis was not properly designed to control for MTX impact on Adalimumab response, and the time of MTX exposition was not exclusion criteria for eligible patients. A prospective cohort study, including an a priori defined time of MTX exposition as mandatory criterion, might be helpful to address this issue. The limited number of included patients might also be responsible for the wide variation of concomitant MTX exposition in our population. Notably, a different duration of exposure to MTX can indeed influence antibody formation, thus eventually biasing the result of the peptide investigation. However, in our cohort mean time of MTX exposition between children showing HC3 and LC3 probes and children who did not, was not different, and, according our χ-square analysis, MTX administration did not influence the presence of the two probes in their samples.

In conclusion our research provides evidence that Adalimumab derived peptides HC3 and LC3 can detect a set of ADAbs, which seem to be linked to disease activity and, probably, to the biologic efficacy and response of the drug. The measurement of these ADAbs might be therefore considered as a potential tool in future clinical trials and practice. Moreover, by using a direct solid phase ELISA we overcome the technical and economical limitations seen with SPR-based optical assay and bridging ELISA. Further validation of our results in a larger patient cohort is needed before wider application of our peptide-based assay method.

As chronic rheumatic disorders show large intrinsic variation due to interaction between the natural history of the disease and the multiple actors modulating response to treatment and the risk of immunogenicity, a theranostic approach should be considered as the future model of care. The standard approach does not consider each single patient’s peculiarities and might be not totally feasible in the bench to bedside translational process to which each clinician is looking for. Novel reliable methods are urgently required to guide and support clinical decisions about switching within or between different biologic drugs in challenging and refractory patients.

## Methods

### Selection of peptides and bioinformatics

Primary amino acid sequence of Adalimumab was retrieved from Drugbank (accession number DB00051). Crystal structure of Adalimumab Fab fragment in complex with TNF-α was retrieved from RCSB Protein Data Bank (accession number 3WD5). Primary amino acid sequence analysis and comparison was performed with GPMAW 7.01 (Lighthouse data, Denmark). Crystal structure analysis was performed using PyMOL (DeLano Scientific LLC, Schrödinger, USA).

### Solid phase peptide synthesis and RP-HPLC purification

The selected Adalimumab heavy and light chain peptides were synthesized using microwave assisted SPPS. Microwave‐assisted syntheses were performed on a Liberty Blue Microwave Automated Peptide Synthesizer (CEM Corporation, Matthews, NC, USA), that combines microwave energy with SPPS using the Fmoc/tert‐butyl strategy.

Fmoc-deprotection cycles are respectively of 15 s (75 °C, 155 W) and 30 s (90 °C, 30 W). The resin was then washed four times with DMF (4 mL each) before coupling of each amino acid. Couplings of Arg residues are performed in 1500 s (25 °C, 0 W) and 300 s (75 °C, 30 W). Couplings of Cys and His are performed in 120 s (25 °C, 0 W) and 240 s (50 °C, 35 W). Couplings of other residues are performed in 15 s (90 °C, 170 W) and 110 s (90 °C, 30 W). After Fmoc deprotection of the last amino acid the resin was washed with CH_2_Cl_2_ (10 mL) and the resin dried.

Peptide cleavage (3 h at 20 °C) from the resin and deprotection of the amino acid side chains were performed with a TFA/H_2_O/TIS (95:2.5:2.5 v/v/v) solution. The resin was then washed with TFA and the filtrate partially evaporated. The crude product was precipitated with diethyl ether, collected by centrifugation, dissolved in water and lyophilized.

Peptides were purified by semi-preparative RP-HPLC using a fully automated Waters Preparative System equipped Phenomenex Synergi 4u Fusion-RP C18 (150 × 10 mm) column operated at 4 ml/min at 25 °C. The solvent systems used were: A (0.1% TFA in H_2_O) and B (0.1 % TFA in CH3CN), running gradient systems of A into B as listed in Table 1 under results. Fractions were analyzed by RP-UPLC ESI-MS (Waters Acquity UPLC coupled to a Waters 3100 ESI-SQD MS). All the peptides were obtained with a purity ≥ 90% to be used for ADAbs detection.

### ELISA

The coating buffer conditions, patient serum dilution, secondary conjugated antibody dilution, and incubation times were previously optimized. Peptides were dissolved in coating Buffer (10 mM Na_2_CO_3_, 30 mM NaHCO_3_, pH 9.6 or TRIS 10 mM pH 8), to 10 µg/mL solution, which was dispensed in each well of the 96-well NUNC Maxisorp (Thermofisher, Italy) microplates. Plates were incubated at 4 °C overnight to allow peptide adsorption to the plate surface. Subsequently, plates were washed 3 times (Tecan HydroFlex, Tecan Group AG, Switzerland) with washing buffer (0.9% NaCl, 0.01% Tween 20), and blocked 1 h at room temperature with 100 µl/well of Fetal Bovine Serum (FBS) Buffer (10% FBS in Washing Buffer) to block non-reacted sites in the plate. FBS buffer was removed, and 100 µl/well of diluted sera sample was dispensed (dilution factor 1:100 except 1:50 for HC2). Plates were incubated at 4 °C overnight to allow the binding of anti-Adalimumab antibodies to coated peptides and then washed 3 times with washing buffer. 100 µl/well of secondary anti-human IgG conjugated to phosphatase alkaline diluted in FBS Buffer (1:3000, Sigma-Aldrich, ref.: A3187, Milano, Italy) were dispensed, and plates were incubated 3 h at room temperature. Plates were washed 3 times with washing buffer, then 100 µl/well of substrate solution (1 mg/ml p-nitrophenylphosphate in coating buffer containing 1 mM of MgCl_2_ were dispensed. Plates were incubated for 15–40 min depending on peptides, and then plates were blocked with 50 µL of 1M NaOH. Absorbance at 405 nm of each well was read with a spectrophotometer (Tecan-Sunrise, Tecan Group AG, Switzerland). Antibody levels were calculated as (mean absorbance of triplicates) − (mean absorbance of blank triplicate. All experiments were performed in compliance with current institutional guidelines.

Statistics was performed using GraphPad Prism 6 (Graphpad Software Inc, USA). Spearman correlation was performed not assuming Gaussian data distribution, calculating non-parametric Spearman correlation factors. Confidence intervals were assumed at 95%. T-test was performed as unpaired data set test, not assuming Gaussian distribution and using Welch´s correction, not assuming equal standard deviation. Receiver operator characteristic (ROC) curves were calculated using 95% confidence intervals.

### Patients’ enrollment

This is an observational time-point cohort study. Patients were recruited from the Pediatric Rheumatology Unit of Anna Meyer Children’s University Hospital, Florence, Italy. Patients with JIA diagnosis, fulfilling the ILAR criteria, or patients with idiopathic chronic uveitis were enrolled if treated with Adalimumab for at least 2 months before study inclusion. Treatment with either Adalimumab as monotherapy or Adalimumab and MTX was allowed at the time of the ADAbs determination. All patients received Adalimumab at the dose of 24 mg/m^2^ subcutaneously every other week. MTX was administered weekly at an average dose of 12.5–15 mg/m^2^. Patients older than 18 years at enrollment, those with other chronic diseases, or with prior other biologic treatments were excluded. The study protocol was approved by the Committee on the Ethics of Azienda Ospedaliera Universitaria Meyer and informed consent was obtained in all cases from legally authorized representative/parents of minors (patients and controls) below age 18 years. JIA-patient disease activity was evaluated summing the four components of the (JADAS-10)^33^. Inactive disease was defined as suggested by Consolaro et al.^34^. Uveitis was defined active according SUN criteria definition, and anterior chamber inflammation was considered inactive or controlled if the inflammatory activity was grade 0 cells according to the SUN Working Group grading scheme for anterior chamber activity^35^.

A cohort of eight gender and age matched JIA patients naive to Adalimumab acted as control group to establish the accuracy of the assays.

### Statistical analysis for anti-Adalimumab antibodies—comparisons and clinical correlations

Chi-square test, and Fisher exact test, when appropriate, were used to compare data. Pearson’s and Spearman’s correlation tests were used to determine correlation coefficients for different entered variables: demographic data, clinical features, received treatment and its duration, and serologic profile. All reported *p*-values were two tailed, and a *p* < 0.05 were considered statistically significant. Analyses were performed on SPSS package for MAC, version 26.0 (SPSS).

### Ethics statement

This study was carried out in strict accordance with the international recommendations. The protocol was approved by the Committee on the Ethics of Azienda Ospedaliera Universitaria Meyer.

## Supplementary Information

Supplementary Information 1.
