# Supplementary material for: A peptide-based anti-Adalimumab antibody assay to monitor immune response to biologics treatment in juvenile idiopathic arthritis and childhood chronic non-infectious uveitis
Source: Sci Rep. 2021 Aug 12;11:16393. doi: 10.1038/s41598-021-95920-9 (PMC8360964; doi:10.1038/s41598-021-95920-9)
Supplement: Supplementary file 1 — Supplementary Information 1. [file 41598_2021_95920_MOESM1_ESM.docx]

**A peptide-based anti-Adalimumab antibody assay to monitor immune response to biologics treatment in juvenile idiopathic arthritis and childhood chronic non-infectious uveitis**

Hendrik Rusche^1§^, Edoardo Marrani^2§^, Feliciana Real-Fernandez^3^, Roberta Ponti^4^, Francesco Terzani^1,3^, Ilaria Maccora^2^, Olivier Monasson^1^, Maria Vincenza Mastrolia^2^, Elisa Peroni^1^, Ilaria Pagnini^2^, Rolando Cimaz^5^, Anna Maria Papini^1,6^, Gabriele Simonini^2,7*^, Paolo Rovero^3*^

Supplementary Material

| **Peptide Purification Summary** | | | | |
| --- | --- | --- | --- | --- |
| **Abbreviation** | **Molecular Weight [Da]** | **Purification Gradient** | **Purity** | **Retention time [min]** |
| HC 1 | 2.245,98 | 20-40% ACN | >95% | 3.63 |
| HC 2 | 2.264,10 | 10-25% ACN | >95% | 3.08 |
| HC 3 | 2.110,98 | 10-35% ACN | >90% | 3.51 |
| HC 3.1 | 3.083,50 | 10-40% ACN | >90% | 3.34 |
| HC 3.2 | 1.774,85 | 20-50% ACN | >95% | 2.81 |
| HC 3.3 | 1.772,84 | 20-50% ACN | >90% | 2.90 |
| LC 1 | 2.308,21 | 10-25% ACN | >90% | 5.15 |
| LC 2 | 2.071,16 | 10-40% ACN | >90% | 3.61 |
| LC 3 | 2.444,08 | 20-50% ACN | >95% | 3.16 |

**Supplementary Table 1:** Overview of purification strategies for synthetic adalimumab peptides and purification success.


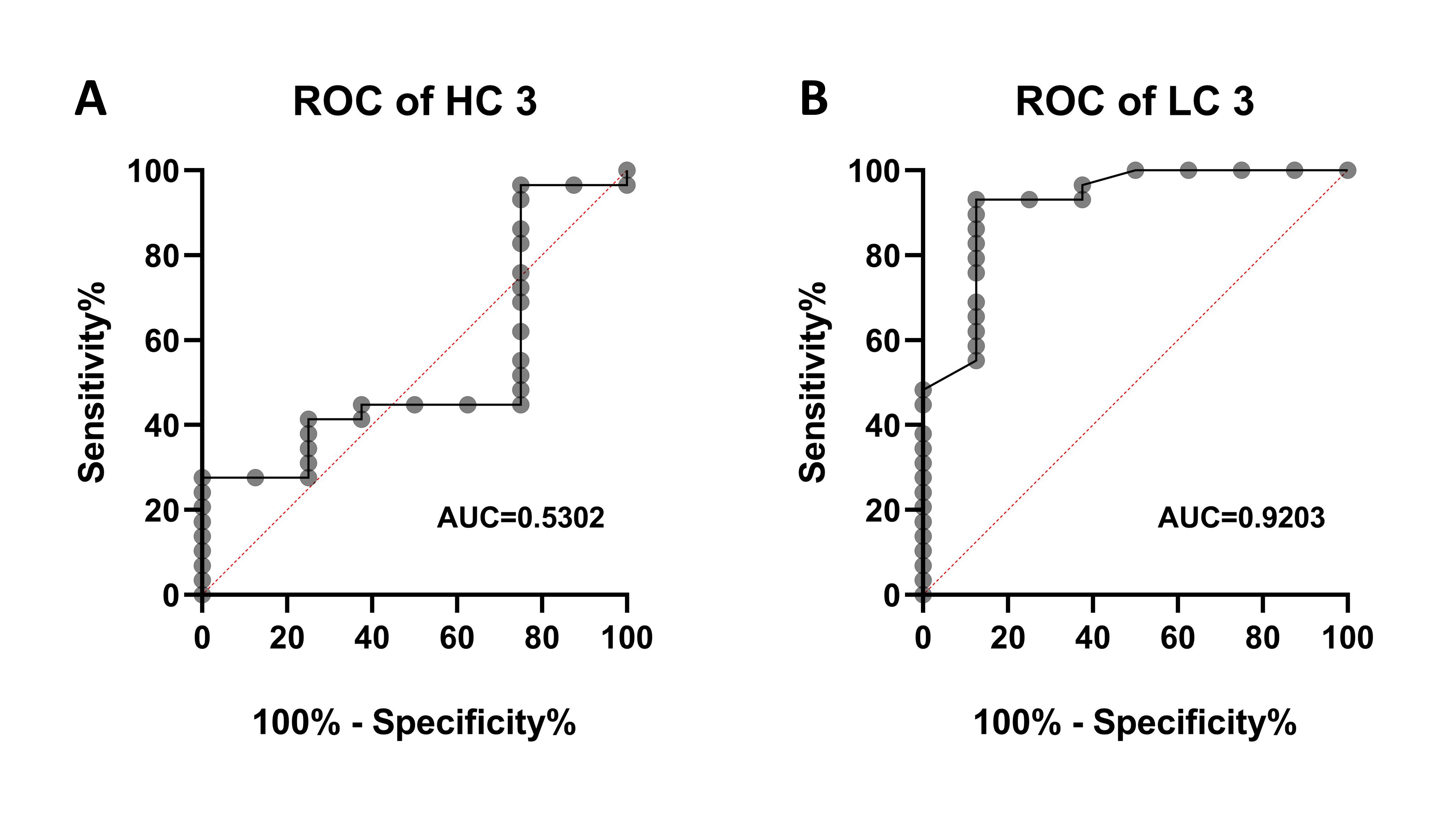


**Supplementary Figure 1.** ROC curve analysis of anti-HC3 (A) and LC3 (B) antibodies in treated patients versus untreated patients determined by ELISA. The calculated area under the curve are plotted in the figures (p<0.0001). Conservatives cut-off values were set up for peptides HC3 and LC3 in 0.2845 [sensitivity 24% (95% CI interval 10.30% to 43.54%) and specificity 100% (95% CI interval 63.06% to 100.0%)] and 0.187 [sensitivity 31% (95% CI interval 15.28% to 50.83%) and specificity 100% (95% CI interval 63.06% to 100.0%)] respectively.

**
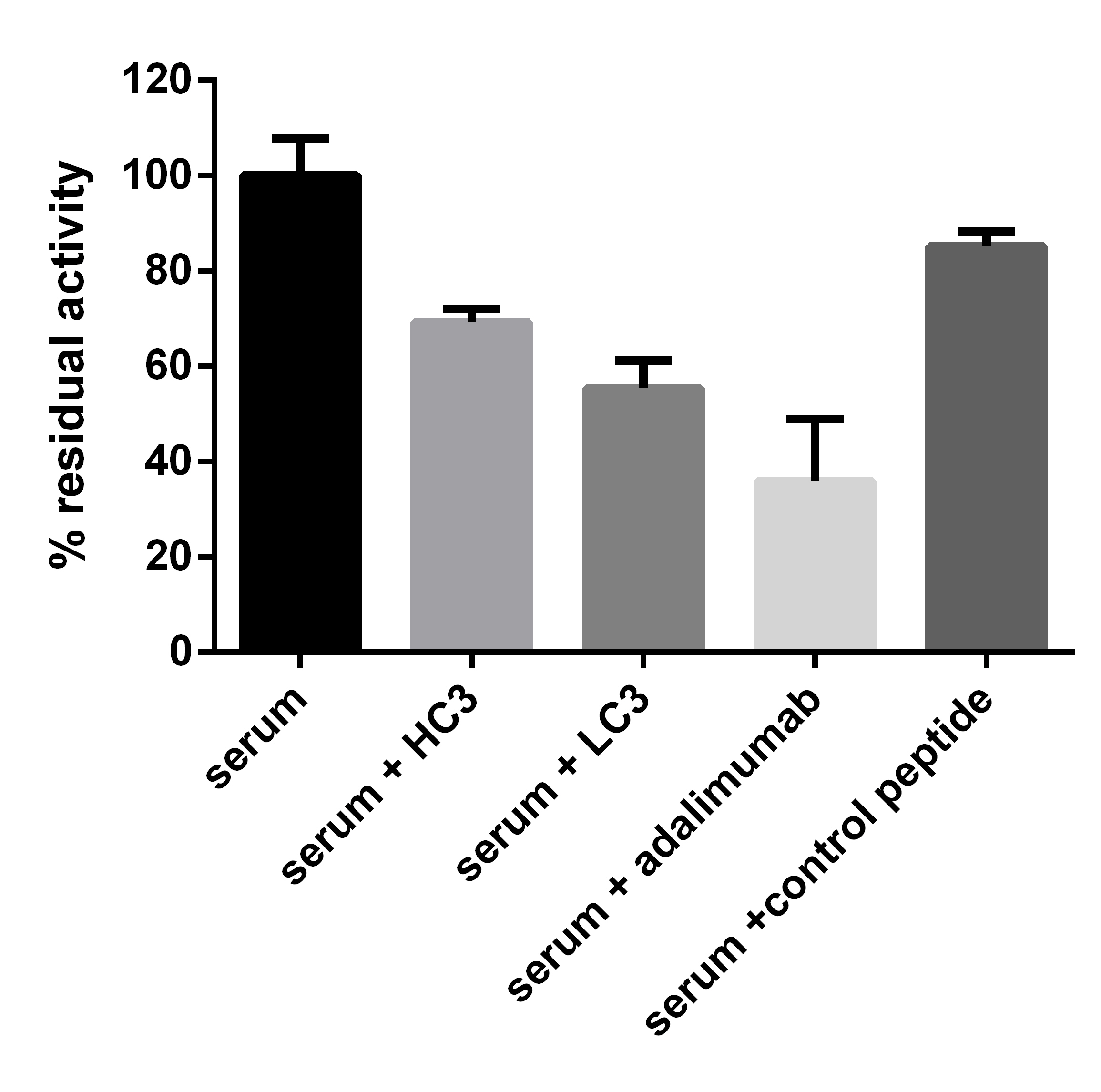
**

**Supplementary Figure 2.** Inhibition experiments of IgG antibodies of a representative serum with peptide HC3, peptide LC3, adalimumab, and a control peptide at concentration 1E-7 M in comparison with the coated peptide HC3 in a competitive ELISA. The results are expressed as the percentage of residual activity of the representative treated patient serum.


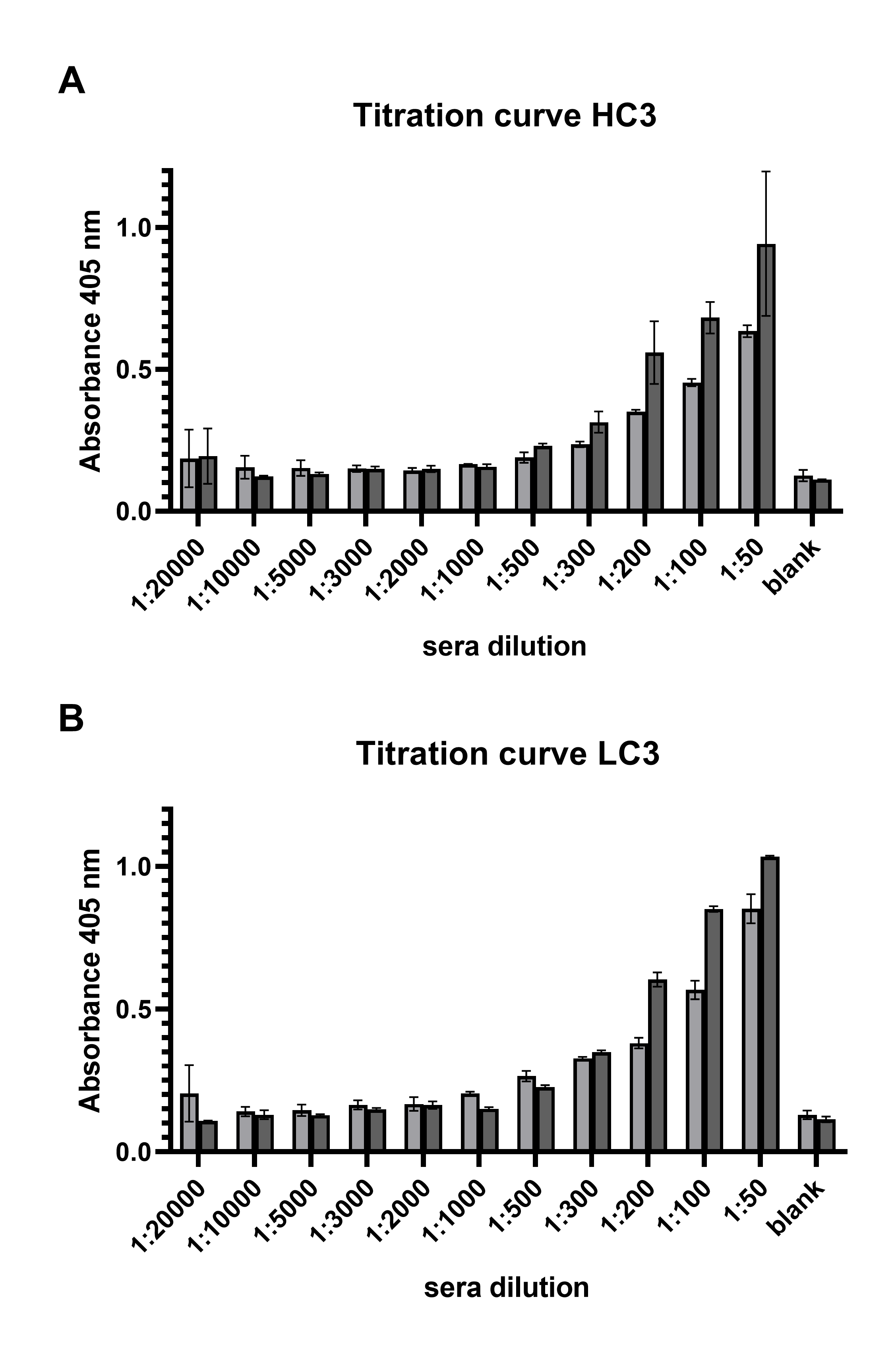


**Supplementary Figure 3:** Antibody titration experiments using (A) HC3 and (B) LC3 were performed following the previously described ELISA conditions using two different sample patient sera added separately at the following serial dilutions: 1:50, 1:100, 1:200, 1:300, 1:500, 1:1000, 1:2000, 1:3000, 1:5000, 1:10000, 1:20000.
